# Supplementary material for: Should we allocate more COVID-19 vaccine doses to non-vaccinated individuals?
Source: PLOS Glob Public Health. 2022 Jul 1;2(7):e0000498. doi: 10.1371/journal.pgph.0000498 (PMC10022372; doi:10.1371/journal.pgph.0000498)
Supplement: S1 Text — Appendix A: Dynamics of the augmented DELPHI model. We describe here the additions made to the DELPHI model to include vaccination states as well as our discretization technique used to enhance the performance of the simulation. Appendix B: Sensitivity Analysis. We explore the sensitivity of our results to key parameters of the model and provide additional simulation results. (PDF) [file pgph.0000498.s001.pdf]

Supplementary material for
Should we allocate more COVID-19 vaccine doses to non-vaccinated individuals?

Zied Ben Chaouch[1,2], Andrew W. Lo[1,2,3,4*], Chi Heem Wong[2,3,4],

1 Department of Electrical Engineering and Computer Science, MIT, Cambridge, MA, United States
2 Laboratory for Financial Engineering, MIT, Cambridge, MA, United States
3 Sloan School of Management, MIT, Cambridge, MA, United States
4 Computer Science and Artificial Intelligence Laboratory, MIT, Cambridge, MA, United States

* alo-admin@mit.edu

A Dynamics of the Augmented DELPHI Model

We review in A.1 the additions made to the DELPHI model introduced in [1] to include vaccination states. We then describe in A.2 our discretization technique to maintain the performance of the model while greatly reducing the running time of the simulation.

A.1 Dynamics of the DELPHI Model

A.1.1 The Original DELPHI Model

The DELPHI model (represented by the green nodes in Fig A.1) is composed of the following components [1]: the susceptible (not yet infected) population (S); exposed individuals that have been infected, are not contagious, and are within the incubation period (E); infected individuals that are currently contagious (I); infected individuals that self-quarantine at home but were not tested (UD and UR), where UR corresponds to the individuals that recover, while UD corresponds to individuals that perish; infected individuals that were detected and hospitalized (DHR and DHD), where DHR corresponds to the individuals that recover, while DHD corresponds to individuals that perish; infected individuals that were detected and quarantined at home (DQR and DQD), where DQR corresponds to the individuals that recover, while DQD corresponds to individuals that perish; individuals that recover from the disease and have permanent immunity (R); and individuals that perish from the disease (D).

The following helpful definitions can also be found in [1]: total number of hospitalized cases (TH); total number of detected deaths (DD); total number of detected cases (DT).

The dynamics of the original DELPHI model are outlined in Eq (A.1) to Eq (A.14) [1]:

$$\frac{\mathrm{d}}{\mathrm{d}t}S(t) = -\alpha \cdot \gamma(t) \cdot S(t) \cdot I(t) \tag{A.1}$$

$$\frac{\mathrm{d}}{\mathrm{d}t}E(t) = \alpha \cdot \gamma(t) \cdot S(t) \cdot I(t) - r_i \cdot E(t) \tag{A.2}$$

$$\frac{\mathrm{d}}{\mathrm{d}t}I(t) = r_i \cdot E(t) - r_d \cdot I(t) \tag{A.3}$$

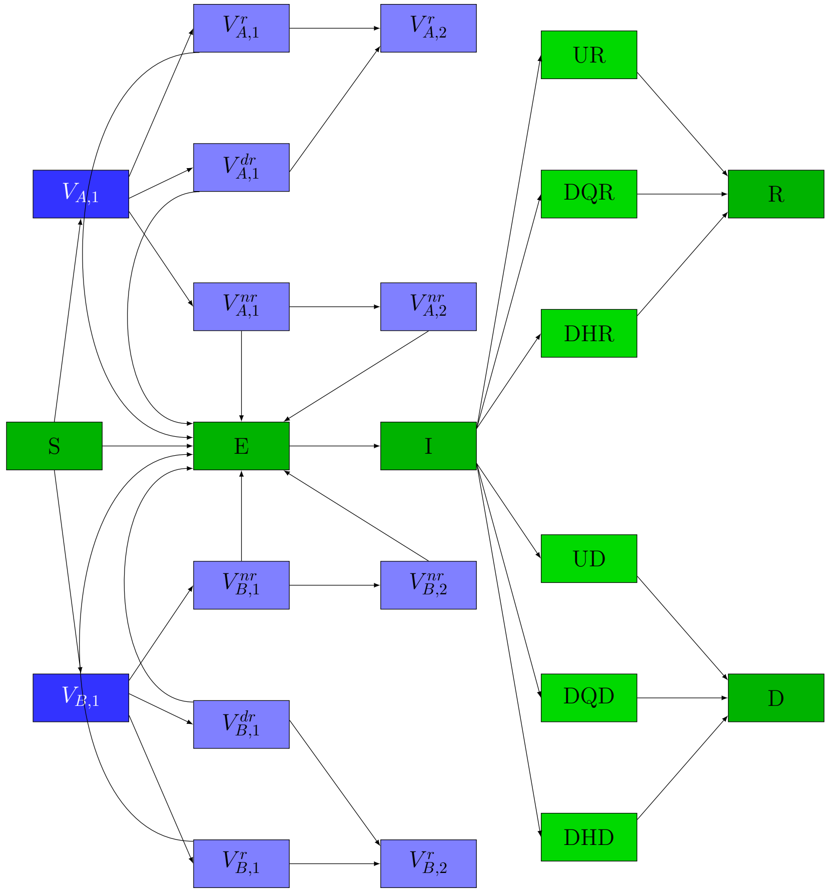

Fig A.1. Flowchart of the original DELPHI model (in green) [1] and the additional vaccination states (in blue) for two hypothetical vaccines. For illustrative purposes, Vaccine A is loosely modelled after the Moderna vaccine, and Vaccine B after the Pfizer-BioNTech vaccine.

$$\frac{\mathrm{d}}{\mathrm{d}t}UR(t) = r_d \cdot (1 - p_{dth}(t)) \cdot (1 - p_d) \cdot I(t) - r_{ri} \cdot UR(t) \tag{A.4}$$

$$\frac{\mathrm{d}}{\mathrm{d}t}DHR(t) = r_d \cdot (1 - p_{dth}(t)) \cdot p_d \cdot p_h \cdot I(t) - r_{rh} \cdot DHR(t) \tag{A.5}$$

$$\frac{\mathrm{d}}{\mathrm{d}t}DQR(t) = r_d \cdot (1 - p_{dth}(t)) \cdot p_d \cdot (1 - p_h) \cdot I(t) - r_{ri} \cdot DQR(t) \tag{A.6}$$

$$\frac{\mathrm{d}}{\mathrm{d}t}UD(t) = r_d \cdot p_{dth}(t) \cdot (1 - p_d) \cdot I(t) - r_{dth} \cdot UD(t) \tag{A.7}$$

$$\frac{\mathrm{d}}{\mathrm{d}t}DHD(t) = r_d \cdot p_{dth}(t) \cdot p_d \cdot p_h \cdot I(t) - r_{dth} \cdot DHD(t) \tag{A.8}$$

$$\frac{\mathrm{d}}{\mathrm{d}t}DQD(t) = r_d \cdot p_{dth}(t) \cdot p_d \cdot (1 - p_h) \cdot I(t) - r_{dth} \cdot DQD(t) \tag{A.9}$$

$$\frac{\mathrm{d}}{\mathrm{d}t}TH(t) = r_d \cdot p_d \cdot p_h \cdot I(t) \tag{A.10}$$

$$\frac{\mathrm{d}}{\mathrm{d}t}DD(t) = r_{dth} \cdot (DHD(t) + DQD(t)) \tag{A.11}$$

$$\frac{\mathrm{d}}{\mathrm{d}t}DT(t) = r_d \cdot p_d \cdot I(t) \tag{A.12}$$

$$\frac{\mathrm{d}}{\mathrm{d}t}R(t) = r_{ri} \cdot (UR(t) + DQR(t)) + r_{rh} \cdot DHR(t) \quad \text{(A.13)}$$

$$\frac{\mathrm{d}}{\mathrm{d}t}D(t) = r_{dth} \cdot (UD(t) + DQD(t) + DHD(t)) \quad \text{(A.14)}$$

Model Calibration To calibrate the DELPHI model, we need to estimate the following quantities [1]:

– α: Infection rate, assumed to be constant across all countries.

– $\gamma(t)$: Government response, modelled as

$$\gamma(t) = \frac{2}{\pi} \cdot \arctan\left(-\frac{b \cdot (t-a)}{20}\right) + 1 + j_0 \cdot \exp\left(-\frac{(t-t_{jump})^2}{2 \cdot \sigma^2}\right), \quad \text{(A.15)}$$

where a controls the time at which the measure starts; b controls the strength of the measure; j_0 controls the magnitude of the jump; t_{jump} is the median day when the jump occurs; and σ is the rate at which the resurgence in the cases occurred.

– r_i: Rate of infection leaving the incubation phase. Assumption: $r_i = \frac{\log 2}{T_i}$, where $T_i = 5$ days (the median time to leave incubation).

– r_d: Rate of detection. Assumption: $r_d = \frac{\log 2}{T_d}$, where $T_d = 2$ days (the median time to detection).

– r_{ri}: Rate of recovery not under hospitalization. Assumption: $r_{ri} = \frac{\log 2}{T_{ri}}$, where $T_{ri} = 10$ days (the median time to recovery not under hospitalization).

– r_{rh}: Rate of recovery under hospitalization. Assumption: $r_{rh} = \frac{\log 2}{T_{rh}}$, where $T_{rh} = 15$ days (the median time to recovery under hospitalization).

– r_{dth}: Rate of death. Assumption: $r_{dth} = \frac{\log 2}{T_{dth}}$, where T_{dth} is the time till death for dying patients.

– $p_{dth}(t)$: Mortality percentage over time, modelled as the following declining function (reflecting an improved ability to detect milder cases and increased standards of care for COVID-19 patients)

$$p_{dth}(t) = (p_{dth0} - \underline{p_{dth}}) \cdot \left[\frac{2}{\pi} \cdot \arctan\left(-\frac{t}{20} \cdot r_{ddec}\right) + 1\right] + \underline{p_{dth}}, \quad \text{(A.16)}$$

where p_{dth0} is the initial mortality percentage; $\underline{p_{dth}}$ is a lower bound on mortality percentage assuming perfect detection and perfect treatment; r_{ddec} is the rate of decay of mortality percentage.

– p_d: Percentage of detected infection cases. Assumption: $p_d = 0.2$ is constant.

– p_h: Percentage of hospitalized detected infection cases. Assumption: $p_h = 0.03$ is constant.

Parameters to Fit The only parameters we need to fit to historical data are:

1. α
2. $r_{dth} = \frac{\log 2}{T_{dth}}$
3. p_{dth0}
4. r_{ddec}
5. a
6. b
7. j_0

8. t_{jump}
9. σ
10. k_1
11. k_2

where k_1 and k_2 are “internal” parameters used for initial conditions.

A.1.2 Inclusion of Vaccination States

In addition to the DELPHI model (represented by green nodes in Fig A.1), we add the following components to account for vaccination states:

- $X \in \{A, B\}$: Brand of the vaccine distributed.
- $V_{X,1}$: Individuals receiving the first dose for vaccine X.
- $V_{X,1}^{r}$ (immediate response): Individuals who respond to the first dose of vaccine X. We assume they have permanent immunity to the disease after a period of $T_{X,I}$ days after receiving the first dose.
- $V_{X,1}^{dr}$ (delayed response): Individuals who do not respond to the first dose of vaccine X but who respond to the second dose of vaccine X. We assume they have permanent immunity to the disease after a period of $T_{X,I}$ days after receiving the second dose.
- $V_{X,1}^{nr}$ (no response): Individuals who do not respond to the first and second doses of vaccine X.

We also need the following helpful definitions:

- $V_{X,a}(t)$: Number of vaccines X available at time t.
- $\varepsilon_{X,i}$: Effectiveness of ith dose of vaccine X [2].
  - Pfizer-BioNTech: 52%efficacy after a single dose, 92%efficacy after two doses.
  - Moderna: 80.20%efficacy after a single dose, 95.60%efficacy after two doses.
- $V^{S}(t)$: Susceptible vaccinated individuals. We assume:

$$V^{S}(t) = \sum_{X \in \{A,B\}} V_{X,1}^{r}(t) + V_{X,1}^{dr}(t) + V_{X,1}^{nr}(t) + V_{X,2}^{nr}(t). \tag{A.17}$$

- $V_{X,2}^{r}$: Individuals who respond to the first and/or second doses of vaccine X, and who were not infected in the first $T_{X,I}$ days following their successful vaccination date.
- $V_{X,2}^{nr}$: Individuals who have still not responded to the second dose of vaccine X after $T_{X,I}$ days following their second vaccination date.
- $V_{X,1r \to 2r}$: individuals that were in the $V_{X,1}^{r}$ state and become $V_{X,2}^{r}$ when they receive their second dose.
- $V_{X,1dr \to 2r}$: individuals that were in the $V_{X,1}^{dr}$ state and become $V_{X,2}^{r}$ when they receive their second dose.
- $V_{X,1nr \to 2nr}$: individuals that were in the $V_{X,1}^{nr}$ state and become $V_{X,2}^{nr}$ when they receive their second dose.

Assumptions In addition, we make the following assumptions:

1. The immune response to a vaccine does not decay over time.
2. All vaccinated individuals receive two doses.
3. Individuals in the $V^r_{X,1}$ group are still susceptible to an infection in the first $T_{X,I}$ days of their first vaccination. Starting on day $T_{X,I}+1$, they have permanent immunity to the disease and join the $V^r_{X,2}$ group.
4. Individuals in the $V^{dr}_{X,1}$ group are still susceptible to an infection until day $T_{X,I}$ following their second vaccination. Starting on day $T_{X,I}+1$ since they receive the second dose, they have permanent immunity to the disease.
5. Individuals in the $V^{nr}_{X,2}$ group will never respond positively to the vaccine, and remain susceptible to an infection.
6. We assume $T_{X,I} = 14$ days [3].
7. We assume a uniform daily infection rate among individuals in each vaccination state.

A.1.3 Dynamics of the DELPHI Model with Vaccination States

We augment the dynamics of the DELPHI model with vaccination states, the blue terms appearing in Eq (A.18) to Eq (A.36):

$$\frac{\mathrm{d}}{\mathrm{d}t}S(t) = -\alpha\cdot\gamma(t)\cdot S(t)\cdot I(t) - \sum_{X\in\{A,B\}} V_{X,1}(t) \tag{A.18}$$

$$\frac{\mathrm{d}}{\mathrm{d}t}E(t) = \alpha\cdot\gamma(t)\cdot(S(t)+V^S(t))\cdot I(t) - r_i\cdot E(t) \tag{A.19}$$

$$\frac{\mathrm{d}}{\mathrm{d}t}V^r_{X,1}(t) = \varepsilon_{X,1}\cdot V_{X,1}(t) - \alpha\cdot\gamma(t)\cdot V^r_{X,1}\cdot I(t) - V_{X,1r\to 2r} \tag{A.20}$$

$$\frac{\mathrm{d}}{\mathrm{d}t}V^{dr}_{X,1}(t) = (\varepsilon_{X,2}-\varepsilon_{X,1})\cdot V_{X,1}(t) - \alpha\cdot\gamma(t)\cdot V^{dr}_{X,1}\cdot I(t) - V_{X,1dr\to 2r} \tag{A.21}$$

$$\frac{\mathrm{d}}{\mathrm{d}t}V^{nr}_{X,1}(t) = (1-\varepsilon_{X,2})\cdot V_{X,1}(t) - \alpha\cdot\gamma(t)\cdot V^{nr}_{X,1}\cdot I(t) - V_{X,1nr\to 2nr} \tag{A.22}$$

$$\frac{\mathrm{d}}{\mathrm{d}t}V^r_{X,2}(t) = V_{X,1r\to 2r} + V_{X,1nr\to 2r} \tag{A.23}$$

$$\frac{\mathrm{d}}{\mathrm{d}t}V^{nr}_{X,2}(t) = V_{X,1nr\to 2nr} - \alpha\cdot\gamma(t)\cdot V^{nr}_{X,2}\cdot I(t) \tag{A.24}$$

$$\frac{\mathrm{d}}{\mathrm{d}t}I(t) = r_i\cdot E(t) - r_d\cdot I(t) \tag{A.25}$$

$$\frac{\mathrm{d}}{\mathrm{d}t}UR(t) = r_d\cdot(1-p_{dth}(t))\cdot(1-p_d)\cdot I(t) - r_{ri}\cdot UR(t) \tag{A.26}$$

$$\frac{\mathrm{d}}{\mathrm{d}t}DHR(t) = r_d\cdot(1-p_{dth}(t))\cdot p_d\cdot p_h\cdot I(t) - r_{rh}\cdot DHR(t) \tag{A.27}$$

$$\frac{\mathrm{d}}{\mathrm{d}t}DQR(t) = r_d\cdot(1-p_{dth}(t))\cdot p_d\cdot(1-p_h)\cdot I(t) - r_{ri}\cdot DQR(t) \tag{A.28}$$

$$\frac{\mathrm{d}}{\mathrm{d}t}UD(t) = r_d\cdot p_{dth}(t)\cdot(1-p_d)\cdot I(t) - r_{dth}\cdot UD(t) \tag{A.29}$$

$$\frac{\mathrm{d}}{\mathrm{d}t}DHD(t) = r_d\cdot p_{dth}(t)\cdot p_d\cdot p_h\cdot I(t) - r_{dth}\cdot DHD(t) \tag{A.30}$$

$$\frac{\mathrm{d}}{\mathrm{d}t}DQD(t) = r_d\cdot p_{dth}(t)\cdot p_d\cdot(1-p_h)\cdot I(t) - r_{dth}\cdot DQD(t) \tag{A.31}$$

$$\frac{\mathrm{d}}{\mathrm{d}t}TH(t) = r_d \cdot p_d \cdot p_h \cdot I(t) \tag{A.32}$$

$$\frac{\mathrm{d}}{\mathrm{d}t}DD(t) = r_{dth} \cdot (DHD(t) + DQD(t)) \tag{A.33}$$

$$\frac{\mathrm{d}}{\mathrm{d}t}DT(t) = r_d \cdot p_d \cdot I(t) \tag{A.34}$$

$$\frac{\mathrm{d}}{\mathrm{d}t}R(t) = r_{ri} \cdot (UR(t) + DQR(t)) + r_{rh} \cdot DHR(t) \tag{A.35}$$

$$\frac{\mathrm{d}}{\mathrm{d}t}D(t) = r_{dth} \cdot (UD(t) + DQD(t) + DHD(t)) \tag{A.36}$$

A.2 Discretization of the DELPHI Model

We simulate the DELPHI model using a time step of 0.01 days. Although these simulations provide a very accurate solution to the DELPHI's system of ODEs, it is not a very practical approach. In fact, running a single simulation for the United States takes a few minutes, which is not an ideal setting for Monte Carlo simulation. To resolve this issue, we recalibrate our parameters and ensure that a discretized version of the DELPHI that uses these parameters and a time step of 1 day will yield the same output as the original non-discretized model. We plot in Fig A.2 a comparison of DELPHI outputs using continuous time steps and discrete time steps of 1 day. These outputs make us confident that using a discretized DELPHI will not affect our results.

We have observed that the parameters $\gamma(t)$, p_h, p_d, and p_{dth} can be left unchanged; however, it was crucial to re-estimate the parameters α, r_i, r_d, r_{ri}, r_{rh}, and r_{dth}. This can be done by noticing that the DELPHI equations yield the following properties:

$$\tilde{\alpha} = -\frac{1}{\gamma(t)} \cdot \frac{S(t+1) - S(t)}{S(t) \cdot I(t)} \tag{A.37}$$

$$\tilde{r_i} = -\frac{[S(t+1) + E(t+1)] - [S(t) + E(t)]}{E(t)} \tag{A.38}$$

$$\tilde{r_d} = -\frac{[S(t+1) + E(t+1) + I(t+1)] - [S(t) + E(t) + I(t)]}{I(t)} \tag{A.39}$$

$$\tilde{r_{dth}} = \frac{D(t+1) - D(t)}{UD(t) + DQD(t) + DHD(t)} = \frac{DD(t+1) - DD(t)}{DQD(t) + DHD(t)} \tag{A.40}$$

Furthermore, we notice that the discretized versions of r_i and r_d are related to their non-discretized versions through the same proportionality coefficient. We use this proportionality coefficient to estimate the discretized version of r_{rh} and r_{ri} from their non-discretized values. In other words,

$$\tilde{r_{ri}} = \frac{\tilde{r_d}}{r_d} \cdot r_{ri} = \frac{\tilde{r_i}}{r_i} \cdot r_{ri} \tag{A.41}$$

$$\tilde{r_{rh}} = \frac{\tilde{r_d}}{r_d} \cdot r_{rh} = \frac{\tilde{r_i}}{r_i} \cdot r_{rh} \tag{A.42}$$

To verify that p_h, p_d, and p_{dth} are not affected by discretization, we estimate them using:

$$\tilde{p_h} = \frac{TH(t+1) - TH(t)}{DT(t+1) - DT(t)} \tag{A.43}$$

$$\tag{A.44}$$

$$\tilde{p_d} = -\frac{DT(t+1) - DT(t)}{[S(t+1) + E(t+1) + I(t+1)] - [S(t) + E(t) + I(t)]} \tag{A.45}$$

$$\tag{A.46}$$

$$\tilde{p_{dth}} = -\frac{[UD(t+1) + DHD(t+1) + DQD(t+1) + D(t+1)] - [UD(t) + DHD(t) + DQD(t) + D(t)]}{[S(t+1) + E(t+1) + I(t+1)] - [S(t) + E(t) + I(t)]} \tag{A.47}$$

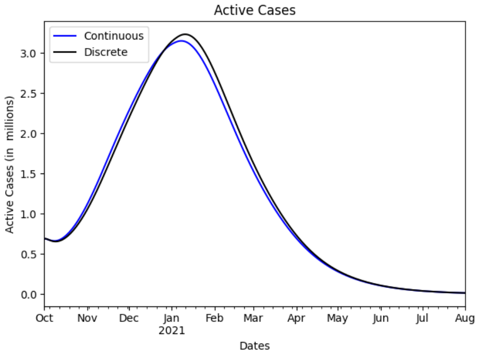

(a) Number of detected cases.

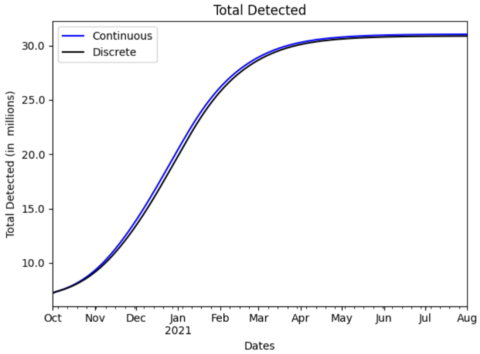

(b) Cumulative number of detected cases.

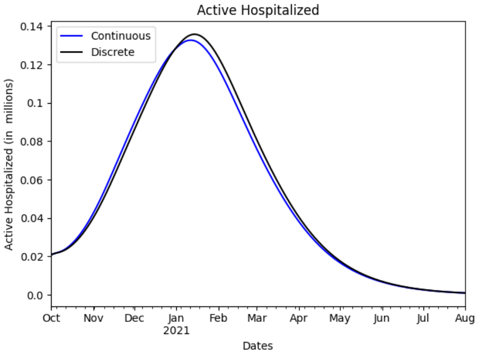

(c) Number of hospitalized cases.

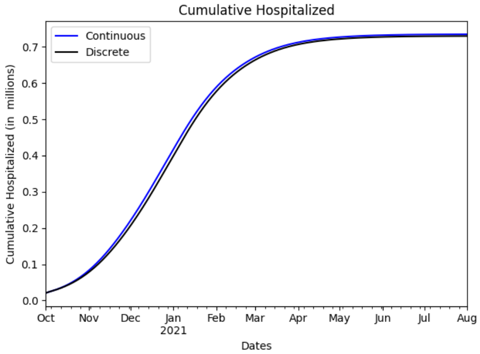

(d) Cumulative number of hospitalized cases.

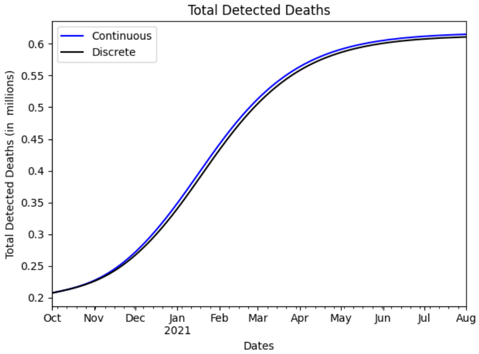

(e) Cumulative number of deaths.

Fig A.2. Comparison of the output for a non-discretized and a discretized (with a time step of 1 day) simulation of the DELPHI. We use the 2021/02/07 DELPHI model parameters.

B Sensitivity Analysis

In this section, we explore the sensitivity of our results to key parameters of the model. In B.1, we delay the second dose by changing the recommended time frame between two doses from 21 days (3 weeks), to 4 weeks, 5 weeks, 7 weeks, and 9 weeks. In B.2, we increase the time to reach permanent immunity after responding positively to a vaccine from 14 days to 21 days. In B.3, we modify the terminal supply rate of vaccines from 1.5 million doses per day to 3.0 million doses per day and 0.75 million doses per day. In B.4, we increase the efficacy of the first dose of the vaccine by 20% and also decrease the efficacy of the first dose of the vaccine by 20%. In B.5, we increase the efficacy of the second dose of the vaccine by 4% and also decrease the efficacy of the first dose of the vaccine by 20%. In B.6, we increase the frequency of supply shocks to 1 per 15 days and also decrease the frequency of supply shocks to 1 per 45 days.

B.1 Impact of Delaying the Second Dose

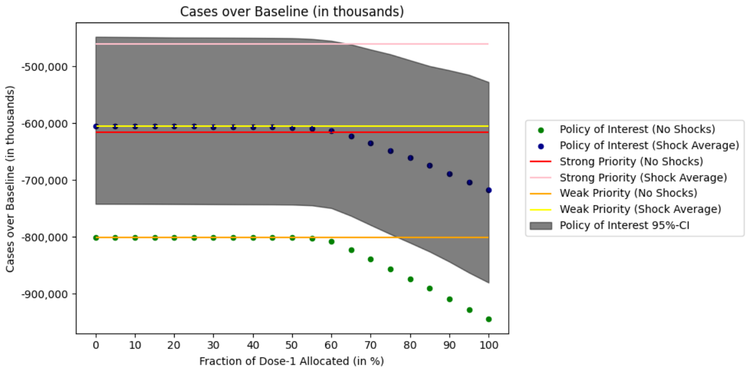

(a) Base Case: 21 days.

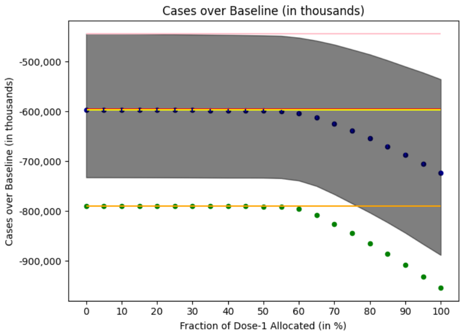

(b) Delay: 28 days.

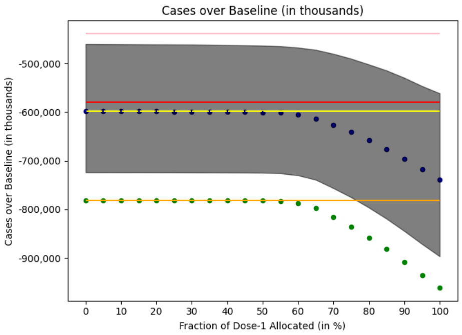

(c) Delay: 35 days.

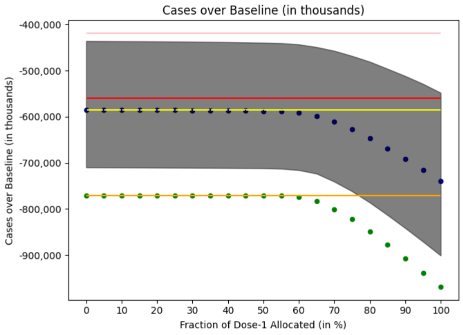

(d) Delay: 49 days.

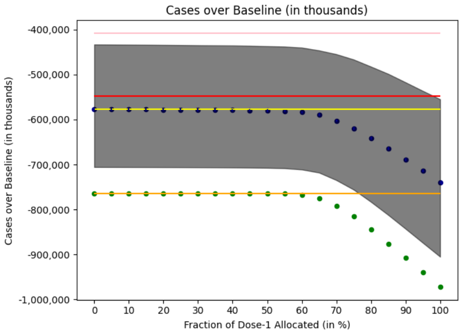

(e) Delay: 63 days.

Fig B.1. Simulation of the DELPHI model under supply shocks. We calculate the **cumulative number of infections** between October 1st, 2020 and August 1st, 2021 relative to a no-vaccination baseline when a constant fraction of available doses are allocated to first-time users. Results under supply shocks are averaged over 1,000 Monte Carlo simulations. We use the February 7th, 2021 DELPHI model parameters.

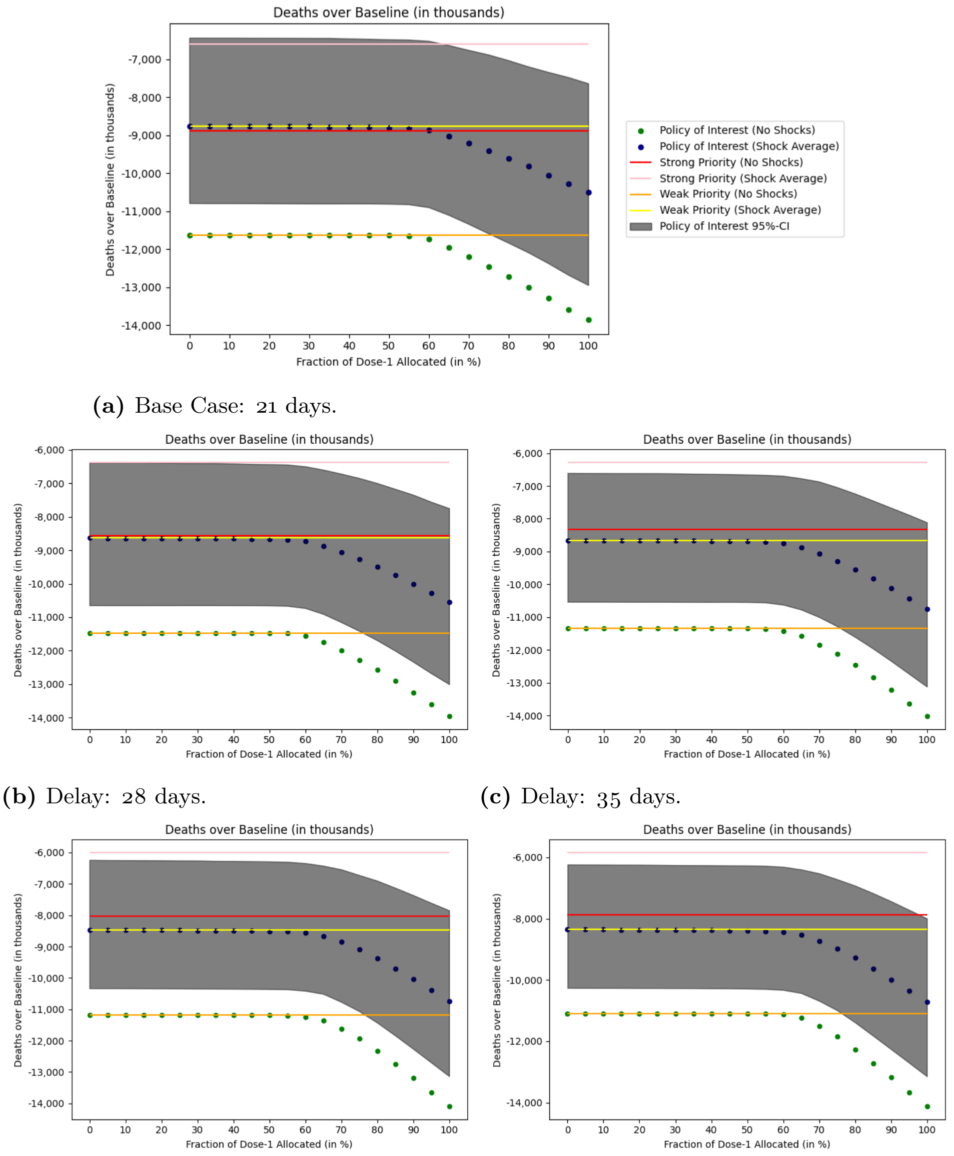

(a) Base Case: 21 days.

(b) Delay: 28 days.

(c) Delay: 35 days.

(d) Delay: 49 days.

(e) Delay: 63 days.

Fig B.2. Simulation of the DELPHI model under supply shocks. We calculate the **cumulative number of deaths** between October 1st, 2020 and August 1st, 2021 relative to a no-vaccination baseline when a constant fraction of available doses are allocated to first-time users. Results under supply shocks are averaged over 1,000 Monte Carlo simulations. We use the February 7th, 2021 DELPHI model parameters.

B.2 Impact of Delaying the Immunity Response

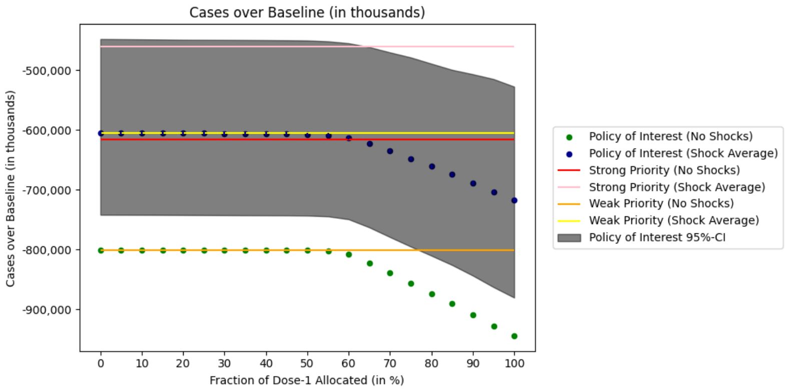

(a) Base Case: 14 days.

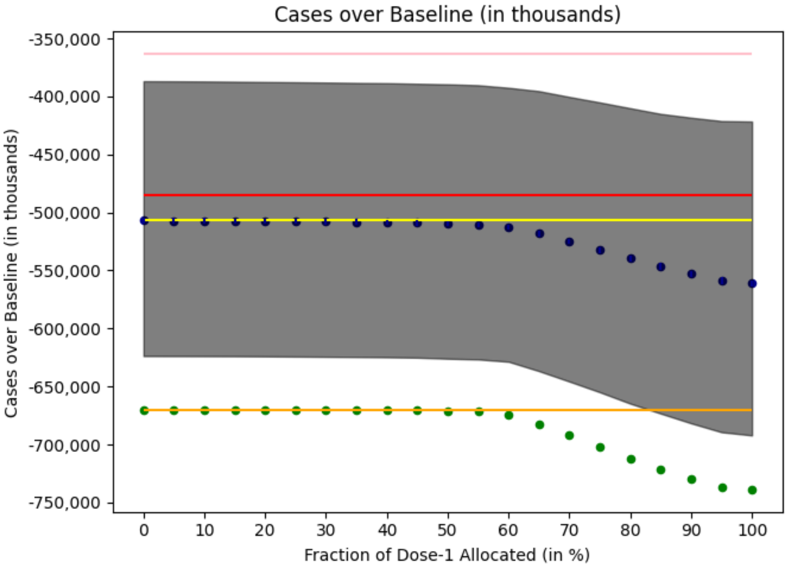

(b) Immunity: 21 days.

Fig B.3. Simulation of the DELPHI model under supply shocks. We calculate the **cumulative number of infections** between October 1st, 2020 and August 1st, 2021 relative to a no-vaccination baseline when a constant fraction of available doses are allocated to first-time users. Results under supply shocks are averaged over 1,000 Monte Carlo simulations. We use the February 7th, 2021 DELPHI model parameters.

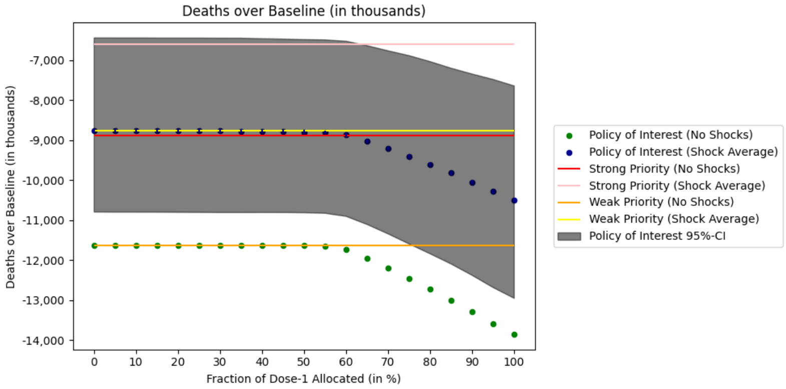

(a) Base Case: 14 days.

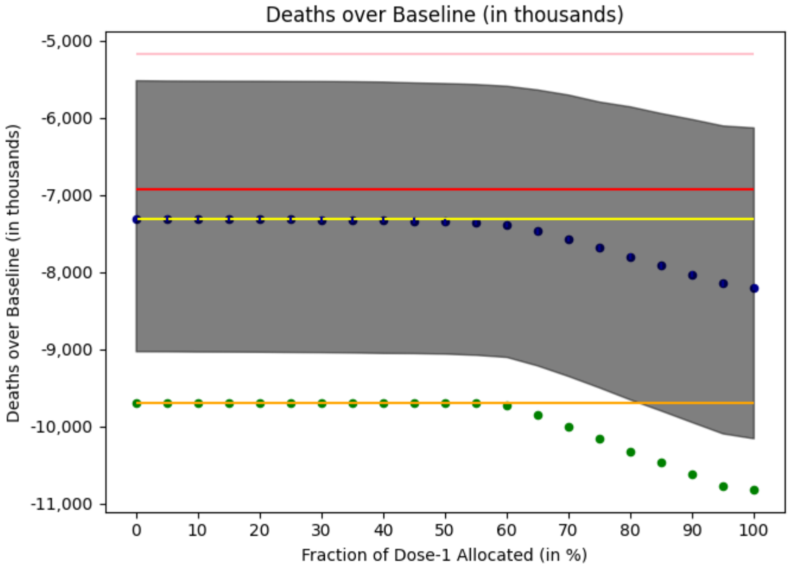

(b) Immunity: 21 days.

Fig B.4. Simulation of the DELPHI model under supply shocks. We calculate the **cumulative number of deaths** between October 1st, 2020 and August 1st, 2021 relative to a no-vaccination baseline when a constant fraction of available doses are allocated to first-time users. Results under supply shocks are averaged over 1,000 Monte Carlo simulations. We use the February 7th, 2021 DELPHI model parameters.

B.3 Impact of the Vaccine's Terminal Supply Rate

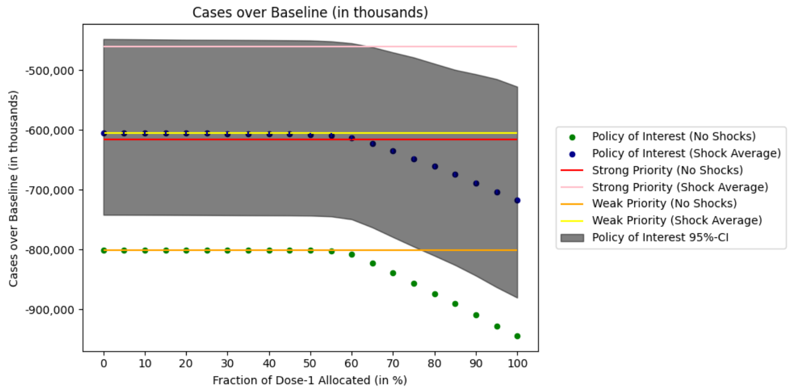

(a) Base Case: 1.5 million per day.

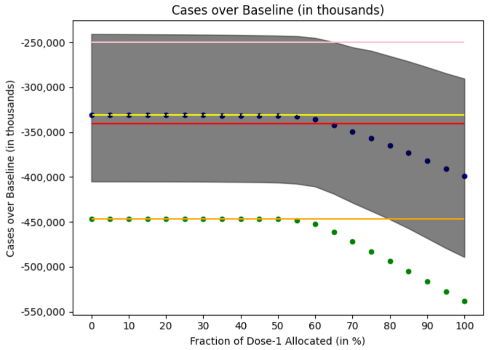

(b) Supply: 0.75 million doses per day.

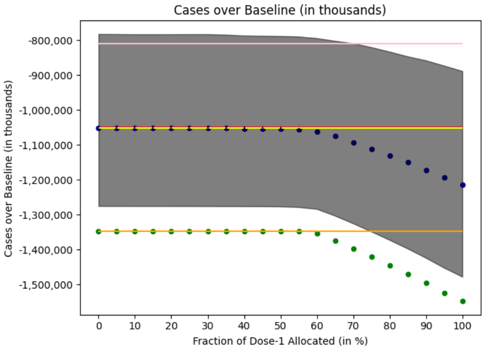

(c) Supply: 3.0 million doses per day.

Fig B.5. Simulation of the DELPHI model under supply shocks. We calculate the **cumulative number of infections** between October 1st, 2020 and August 1st, 2021 relative to a no-vaccination baseline when a constant fraction of available doses are allocated to first-time users. Results under supply shocks are averaged over 1,000 Monte Carlo simulations. We use the February 7th, 2021 DELPHI model parameters.

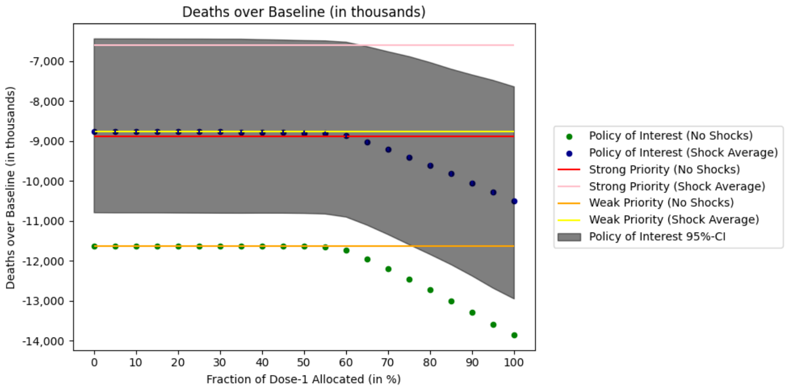

(a) Base Case: 1.5 million per day.

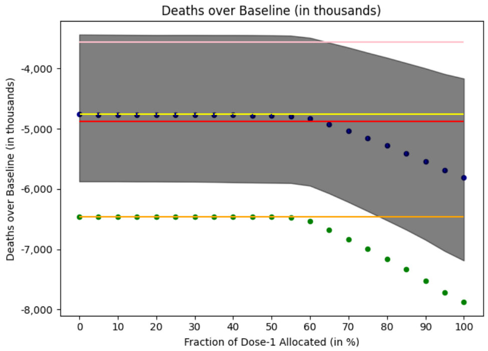

(b) Supply: 0.75 million doses per day.

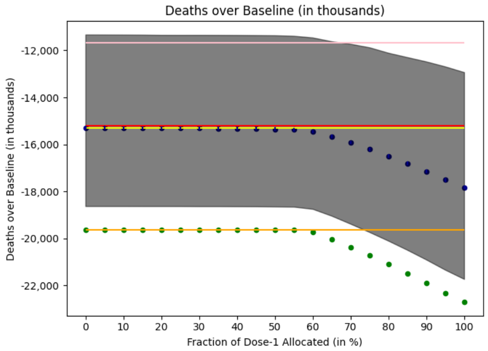

(c) Supply: 3.0 million doses per day.

Fig B.6. Simulation of the DELPHI model under supply shocks. We calculate the **cumulative number of deaths** between October 1st, 2020 and August 1st, 2021 relative to a no-vaccination baseline when a constant fraction of available doses are allocated to first-time users. Results under supply shocks are averaged over 1,000 Monte Carlo simulations. We use the February 7th, 2021 DELPHI model parameters.

B.4 Impact of the First Dose Efficacy

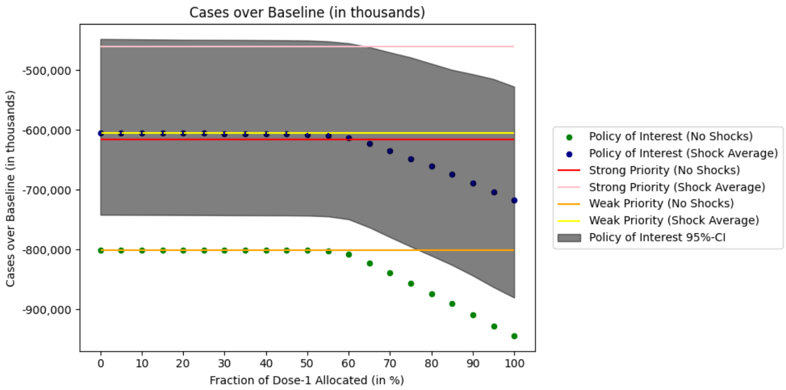

(a) Base Case: 52% (Pfizer), 80.20% (Moderna).

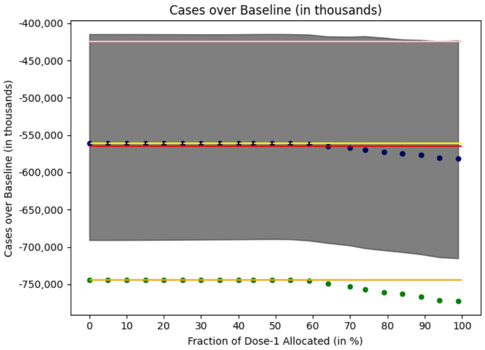

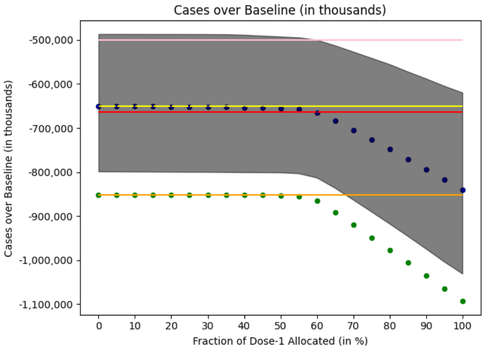

(b) Efficacy: -20%.

(c) Efficacy: +20%.

Fig B.7. Simulation of the DELPHI model under supply shocks. We calculate the **cumulative number of infections** between October 1st, 2020 and August 1st, 2021 relative to a no-vaccination baseline when a constant fraction of available doses are allocated to first-time users. Results under supply shocks are averaged over 1,000 Monte Carlo simulations. We use the February 7th, 2021 DELPHI model parameters.

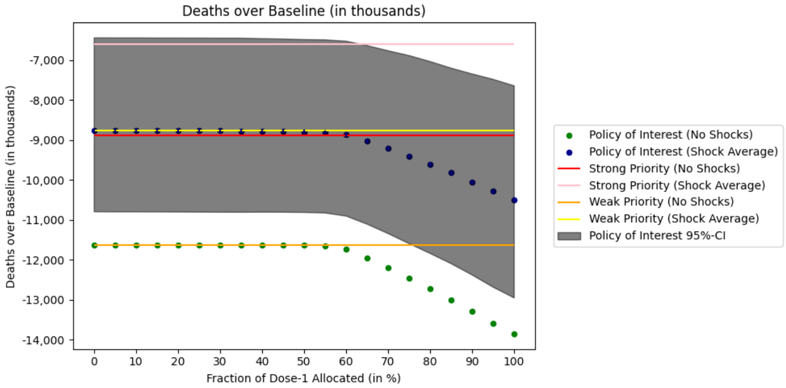

(a) Base Case: 52% (Pfizer), 80.20% (Moderna).

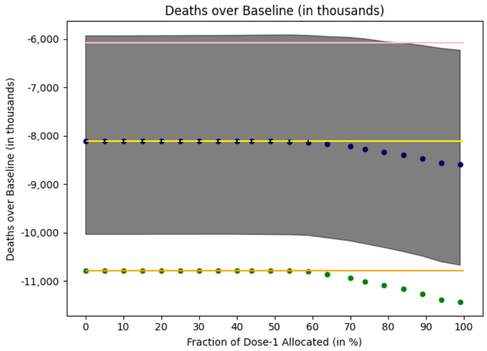

(b) Efficacy: -20%.

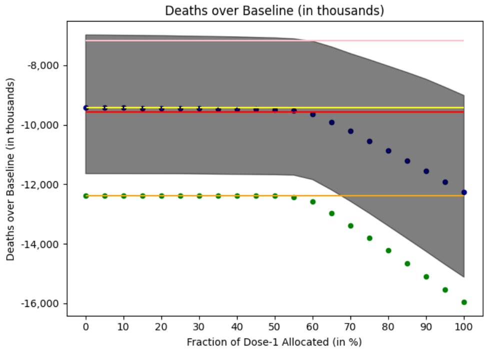

(c) Efficacy: +20%.

Fig B.8. Simulation of the DELPHI model under supply shocks. We calculate the **cumulative number of deaths** between October 1st, 2020 and August 1st, 2021 relative to a no-vaccination baseline when a constant fraction of available doses are allocated to first-time users. Results under supply shocks are averaged over 1,000 Monte Carlo simulations. We use the February 7th, 2021 DELPHI model parameters.

B.5 Impact of the Second Dose Efficacy

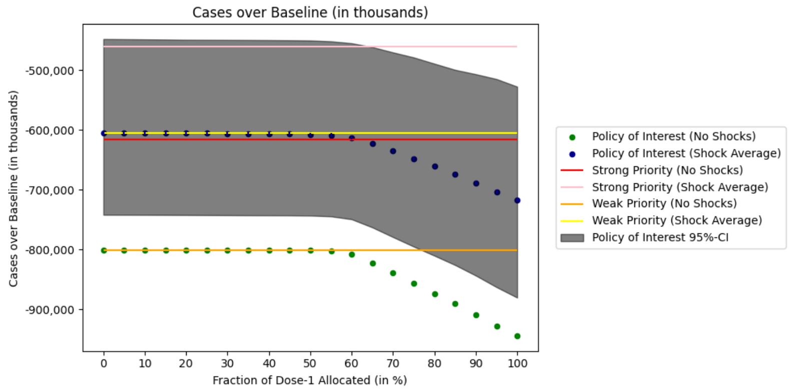

(a) Base Case: 92% (Pfizer), 95.60% (Moderna).

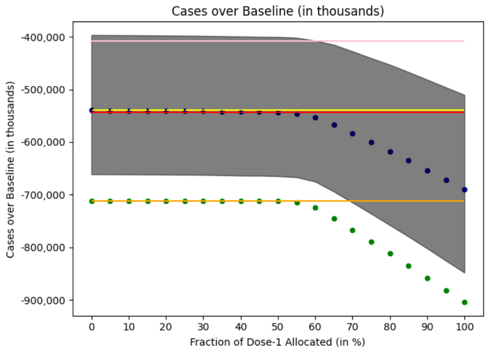

(b) Efficacy: -20%.

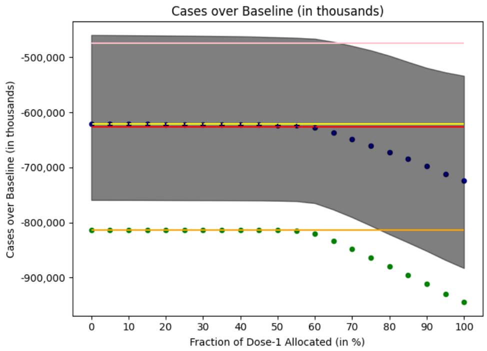

(c) Efficacy: +4%.

Fig B.9. Simulation of the DELPHI model under supply shocks. We calculate the **cumulative number of infections** between October 1st, 2020 and August 1st, 2021 relative to a no-vaccination baseline when a constant fraction of available doses are allocated to first-time users. Results under supply shocks are averaged over 1,000 Monte Carlo simulations. We use the February 7th, 2021 DELPHI model parameters.

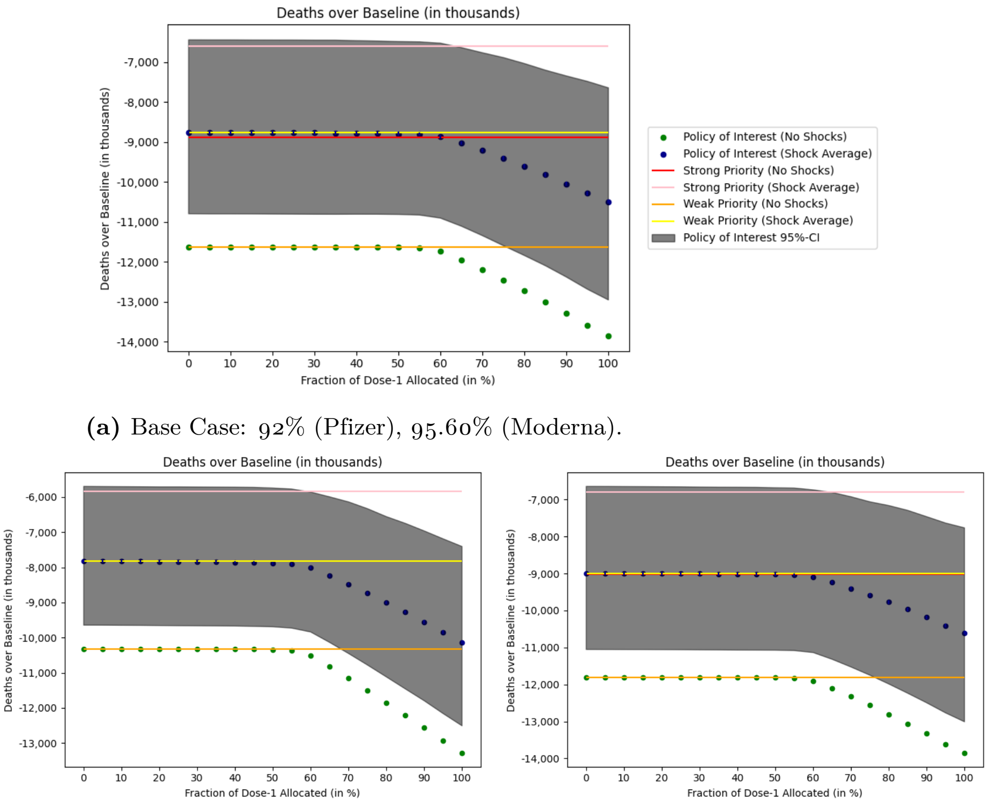

(a) Base Case: 92% (Pfizer), 95.60% (Moderna).

(b) Efficacy: -20%.

(c) Efficacy: +4%.

Fig B.10. Simulation of the DELPHI model under supply shocks. We calculate the **cumulative number of deaths** between October 1st, 2020 and August 1st, 2021 relative to a no-vaccination baseline when a constant fraction of available doses are allocated to first-time users. Results under supply shocks are averaged over 1,000 Monte Carlo simulations. We use the February 7th, 2021 DELPHI model parameters.

B.6 Impact of the Frequency of Supply Shocks

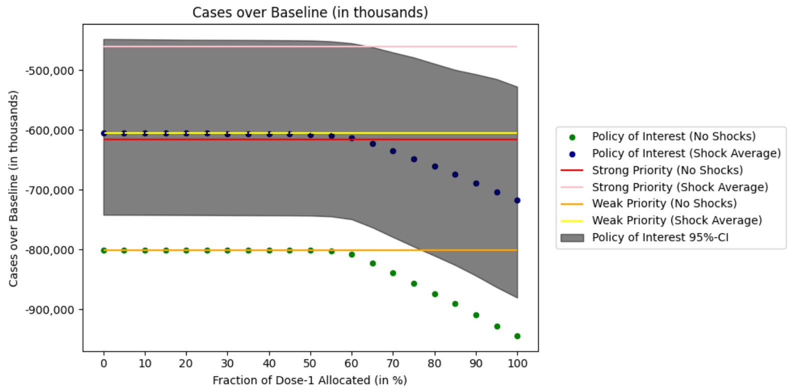

(a) Base Case: 1/30 day^{-1}.

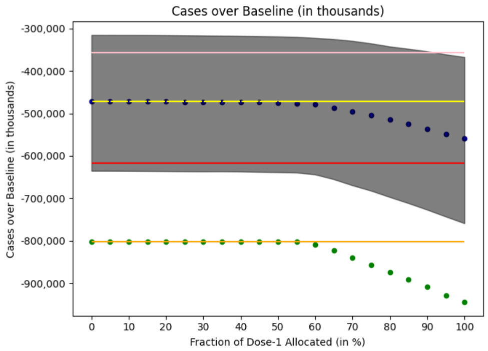

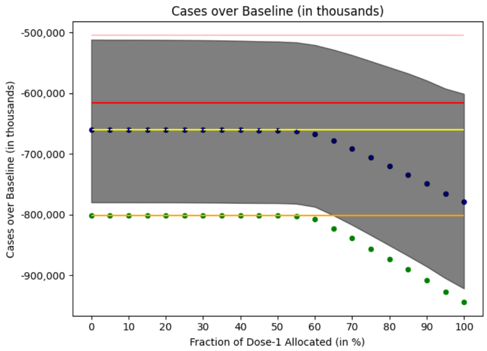

(b) Shock Frequency: 1/15 day^{-1}. **(c)** Shock Frequency: 1/45 day^{-1}.

Fig B.11. Simulation of the DELPHI model under supply shocks. We calculate the **cumulative number of infections** between October 1st, 2020 and August 1st, 2021 relative to a no-vaccination baseline when a constant fraction of available doses are allocated to first-time users. Results under supply shocks are averaged over 1,000 Monte Carlo simulations. We use the February 7th, 2021 DELPHI model parameters.

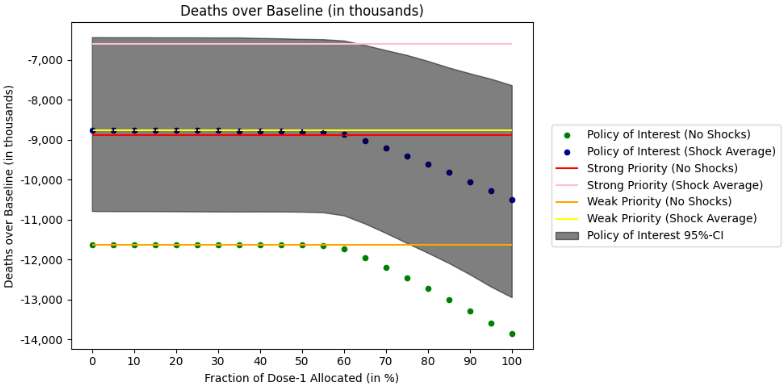

(a) Base Case: $1/30$ day^{-1}.

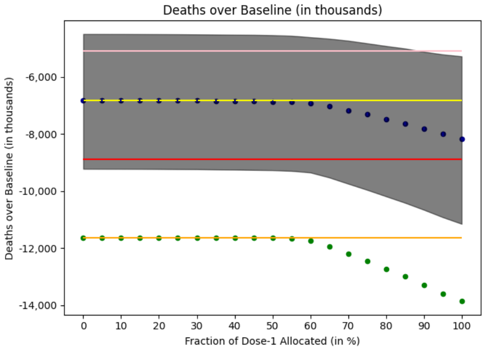

(b) Shock Frequency: $1/15$ day^{-1}.

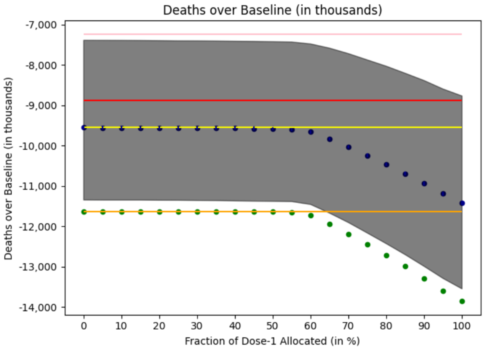

(c) Shock Frequency: $1/45$ day^{-1}.

Fig B.12. Simulation of the DELPHI model under supply shocks. We calculate the **cumulative number of deaths** between October 1st, 2020 and August 1st, 2021 relative to a no-vaccination baseline when a constant fraction of available doses are allocated to first-time users. Results under supply shocks are averaged over 1,000 Monte Carlo simulations. We use the February 7th, 2021 DELPHI model parameters.

References

1. Li M, Bouardi H, Lami O, Trikalinos T, Trichakis N, Bertsimas D. Forecasting COVID-19 and Analyzing the Effect of Government Interventions; 2020.

2. Gorvett Z. How effective is a single vaccine dose against Covid-19?; 2021. Available from: https://www.bbc.com/future/article/20210114-covid-19-how-effective-is-a-single-vaccine-dose.

3. Regalado A. The chart that shows how we'll get back to normal; 2020. Available from: https://www.technologyreview.com/2020/12/10/1013914/pfizer-biontech-vaccine-chart-covid-19/.
