# Supplementary material for: Should we allocate more COVID-19 vaccine doses to non-vaccinated individuals?
Source: PLOS Glob Public Health. 2022 Jul 1;2(7):e0000498. doi: 10.1371/journal.pgph.0000498 (PMC10022372; doi:10.1371/journal.pgph.0000498)
Supplement: Attachment — Submitted filename: Response_to_Reviewers.docx [file pgph.0000498.s002.docx]

# Response to Reviewers

We thank the reviewers and editor for their guidance and assistance. Please find our comments addressing each reviewer’s remarks (in blue) below.

**Reviewer 1:**

Authors have discussed various vaccination distributions policies to have a better effect in COVID control. They analyzed that allocating more than 50% of available doses to individuals who have not received their first dose can significantly increase the number of lives saved and significantly reduce the number of COVID-19 infections relative to a policy that guarantees a second dose within the recommended time frame to all individuals who have already received their first dose.

This is a useful work, and the questions has been analyzed properly. I recommend for acceptance in its current form.

We thank the reviewer for taking the time to read and provide feedback our manuscript.

**Reviewer 2:**

This study on vaccine supply and management is very important study when there has been frequent emergence of variants of Sars-Cov-2 virus due to large proportion of global population still remaining unvaccinated against Covid19. This study generates the evidence for efficiency of vaccination policy. Allocating 50% vaccines for first time users and vaccinating them may significantly reduce the deaths as well spread of Covid19. These results are in concurrence to ongoing discussion and research on partially vaccinating the majority of global population will reduce the emergence of variants.

We thank the reviewer for appreciating the scope of the analysis and providing helpful feedback on our manuscript.

Authors may give reasons for their assumptions such as

- Why DELPHI model is discretized rather than working with ODE (continuous) model (Page 5)

A detailed discussion of the discretized model (and a comparison with the continuous version) is provided in Appendix A2. Discretizing the model dynamics should produce a similar output to the continuous-time version as long as the time step used is “small” (for example 0.01 days has been commonly used in the literature when working with the SEIR model). In our case, we could not afford to use such a fine time step as it would be computationally intensive when running Monte Carlo simulations. This is described in the manuscript (Page 5) in the sentence:

“This step is crucial, as it considerably improves the speed of the simulation and allows us to run Monte Carlo analyses.”

We instead recalibrate the model parameters in order to use a time step of 1 day. In Appendix A2, we show that the recalibration technique works empirically: Figure 6 shows that the outputs from the continuous time version are well approximated by the outputs from our discretized model.

- Choice of Poisson distribution to model vaccine supply shocks (Page 7)

Thank you for pointing this out. We have added the following sentence to the paragraph to justify the use of a Poisson process:

“Using a Poisson process is appropriate here as we assume that shocks that occur over disjoint time intervals are independent, and that the process is memoryless.”

We also explore the sensitivity of the simulation’s outputs to the Poisson rate in Appendix B6.

- Carrying out only 1000 iterations of Monte Carlo simulation (Page 7)

We have explored using 10,000 and 20,000 iterations for the Monte Carlo simulations and the results were consistent with the outputs from the 1,000 iterations simulation. Fundamentally, the randomness comes only from the supply shocks, and using more simulations can help shrink the confidence interval. For computational purposes, given that running 1,000 iterations takes almost an hour (with a parallelized code), it becomes computationally impractical to simulate more than 20,000 iterations. We trade off slightly larger confidence intervals for a faster simulation. However, this does not affect our results as the confidence intervals we obtain from 1,000 iterations are already small enough to detect a significant difference across the policies explored.

To make our point clearer, we have included the following sentence in the manuscript:

“After comparing the outputs obtained with various number of Monte Carlo simulations, we selected the number of Monte Carlo simulations to be large enough to reflect the uncertainty in the output while being parsimonious enough to retain a practical simulation runtime.”

- Shocks lasting 7days or more is assumed to have 50%probability of boosting the terminal supply rate by 5%.

We justify this in the manuscript in the sentence “Supply shocks can simply represent delays in production or delivery of vaccines, but they can also model a factory shutdown aimed at improving the production of vaccines.”

The idea here is that long supply shocks tend to be long for a fundamental reason. This reason could be related to a factory shutdown (e.g., as described in reference [13]), which we assume would increase the terminal rate by 5%. However, this is not a guarantee and so we assume that long shocks would correspond to a factory improvement only 50% of the time. There is no particular reason to choose 5% as a number, however we explore the simulation’s outputs to the terminal supply rate in Appendix B3.

To make our point clearer, we have rephrased it to:

“Supply shocks can simply represent delays in production or delivery of vaccines (which would tend to last a few days), but they can also model a factory shutdown aimed at improving the production of vaccines (which would tend to last longer and may increase the terminal supply rate).”

Giving reason for all these assumptions will be strengthen the study from the point of view of research and will be helpful for the early researchers.

We address each concern raised by the reviewer above.
